# Artificial intelligence-based real-time histopathology of gastric cancer using confocal laser endomicroscopy

**DOI:** 10.1038/s41698-024-00621-x

**Published:** 2024-06-14

**Authors:** Haeyon Cho, Damin Moon, So Mi Heo, Jinah Chu, Hyunsik Bae, Sangjoon Choi, Yubin Lee, Dongmin Kim, Yeonju Jo, Kyuyoung Kim, Kyungmin Hwang, Dakeun Lee, Heung-Kook Choi, Seokhwi Kim

**Affiliations:** 1 Department of Pathology, Asan Medical Center, University of Ulsan College of Medicine, Seoul, Republic of Korea; 2 Artificial Intelligence Research Center, JLK Inc., Seoul, Republic of Korea; 3 Department of Pathology, Ajou University School of Medicine, Suwon, Republic of Korea; 4 Department of Pathology, Kangbuk Samsung Hospital, Sungkyunkwan University School of Medicine, Seoul, Republic of Korea; 5 Department of Pathology and Translational Genomics, Samsung Medical Center, Sungkyunkwan University School of Medicine, Seoul, Republic of Korea; 6 Pathology center, Seegene Medical Foundation, Seoul, Republic of Korea; 7 VPIX Medical Inc., Daejeon, Republic of Korea; 8 Department of Biomedical Sciences, Ajou University Graduate School of Medicine, Suwon, Republic of Korea

**Keywords:** Translational research, Gastric cancer

## Abstract

There has been a persistent demand for an innovative modality in real-time histologic imaging, distinct from the conventional frozen section technique. We developed an artificial intelligence-driven real-time evaluation model for gastric cancer tissue using confocal laser endomicroscopic system. The remarkable performance of the model suggests its potential utilization as a standalone modality for instantaneous histologic assessment and as a complementary tool for pathologists’ interpretation.

Timely histological assessment of fresh tissue during surgical operations is imperative for effective cancer treatment, encompassing the identification and evaluation of tumor cells to inform treatment strategies and ensure surgical resection with adequate margins. Nevertheless, the conventional frozen section technique currently employed for this purpose is associated with inherent limitations, primarily related to processing time and preparation artifacts, rendering it susceptible to errors^1^.

An emerging alternative is the recently introduced confocal laser endomicroscopic system (CLES)^2^. The confocal microscope, traditionally utilized for high-resolution imaging in experimental biology, has undergone recent hardware advancements enabling its miniaturization into a handheld endomicroscopic probe^3^. This portable unit comprises the laser source and signal processing device. Although the CLES has demonstrated its efficacy in real-time histologic imaging across various organs in mice^3^ and human samples, including brain tumors^4^, its application in gastric tissue remains constrained with limited interpretation performance^5–9^. Furthermore, the grayscale images generated by the CLES pose challenges in interpretation, even for experienced pathologists, necessitating considerable time and effort for adaptation. This underscores the pressing need for the development of an automated interpretation system.

Recent strides in artificial intelligence (AI) algorithms have expanded their applications in the medical field^10,11^. In histologic image interpretation, AI models have proven valuable across various cancer types^12–15^, not only for interpreting hematoxylin and eosin (H&E) images but also for analyzing immunohistochemical staining images, which often require rigorous quantification to inform treatment strategies^16^. In the domain of digital pathology AI research, a prevalent approach involves employing AI models to segment gigapixel pathology images into smaller patch images. Subsequently, the AI model is utilized to detect tumor areas within each patch image^17^. Alternatively, a whole-slide-image-level diagnosis can be achieved through the implementation of weakly supervised learning models^18^. As these methodologies continue to evolve, the comprehensive validation of AI models for interpretation becomes imperative, particularly when considering their deployment as standalone modalities^19^.

In this study, we developed an AI model tailored for the real-time and automated detection of cancer cells within CLES images. The devised two-stage AI model comprises a decisive phase discerning between tumor and normal images, followed by histologic subtyping stages. Our findings reveal that the model demonstrates remarkable proficiency in determining the presence of tumor when compared to assessments by pathologists. Moreover, the integration of AI assistance in the interpretation of CLES images by pathologists markedly augmented their diagnostic capabilities. This study underscores the potential of our AI model for real-time gastric cancer detection in CLES images, showcasing its versatility for utilization as both a standalone modality and a tool to enhance pathologists’ image interpretation proficiency.

The hardware components of the utilized CLES in this study comprised the light source, an image processor, and a maneuverable endomicroscopic head controlled by a joy-stick-like device (Fig. 1a, b). Upon specimen placement on the device, real-time imaging of the tissue was promptly achievable through laser transmission. The acquired image instantaneously appeared on the monitor, accompanied by the AI model’s prompt interpretation of cancer probability, which could be visualized after the completion of the imaging (Fig. 1c, Supplementary Fig. 1, and Supplementary Video 1). CLES images obtained from non-neoplastic gastric tissue distinctly portrayed the characteristics of each layer – mucosa, submucosa, and muscularis propria (Fig. 1d and Supplementary Fig. 2a). In mucosal layer imaging, the CLES images effectively depicted the round or ovoid architecture of glands. Within the submucosal layer, the loose fibrotic structure was discernible in the CLES images. In the muscularis propria, the CLES images illustrated the arrangement of muscle fibers. Tumor cells were conspicuously evident in CLES images (Fig. 1e and Supplementary Fig. 2b). Tubular adenocarcinoma, a histologic subtype of gastric cancer resembling the original architecture of gastric mucosal glands, manifested as bright round or ovoid objects in the CLES images. Conversely, poorly cohesive carcinoma, another subtype of gastric cancer characterized by the diffuse, scattered distribution of individual cancer cells, was portrayed as scattered small bright dots in CLES images, recapitulating its histomorphology in H&E stains.

**Fig. 1:**
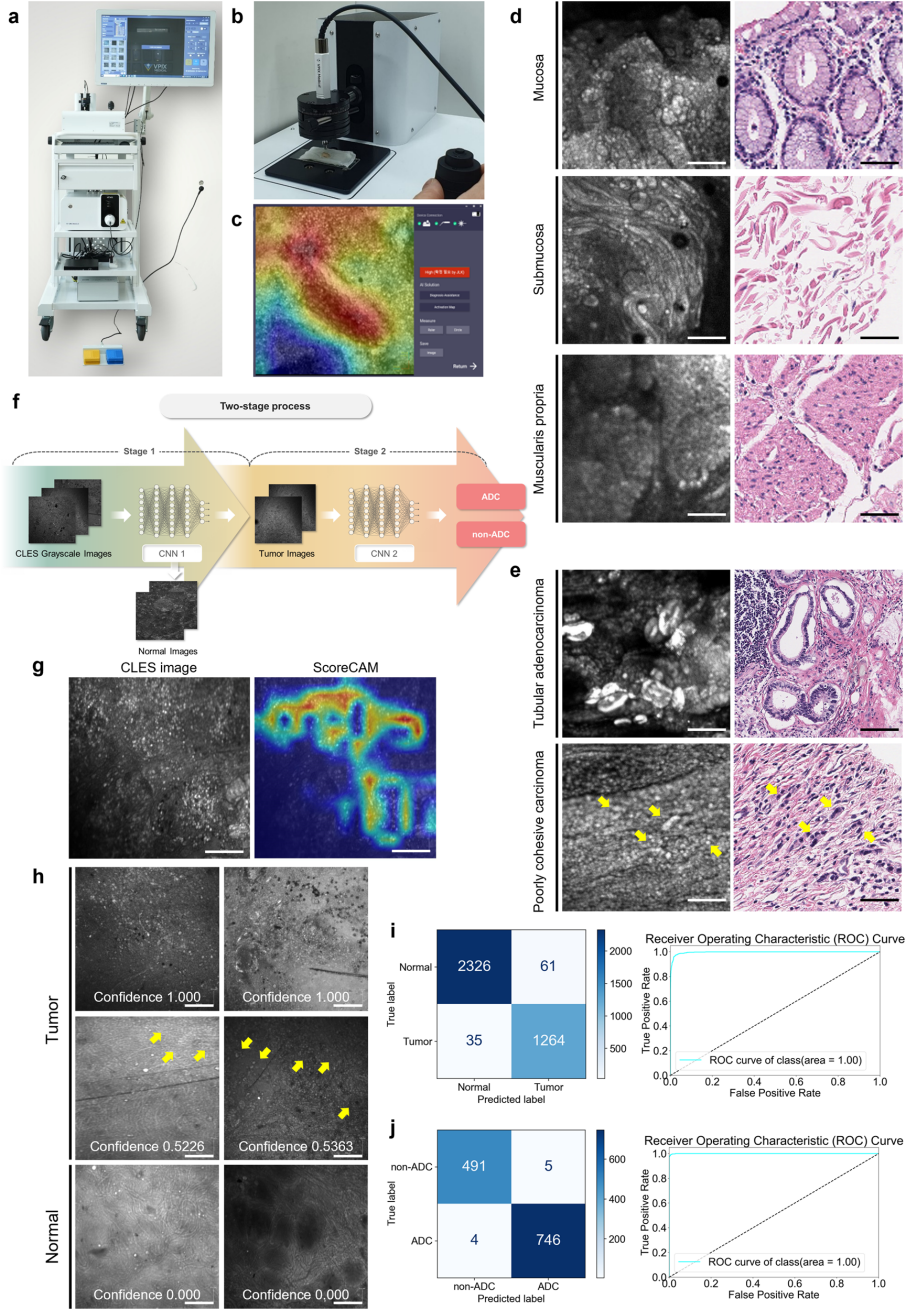
Development of an artificial intelligence-based confocal laser endomicroscopic system. **a** The hardware component of the confocal laser endomicroscopic system (CLES). Reprinted with permission from VPIX Medical, Inc. **b** Elaboration on the microscopic head and controller components. **c** Illustration of a CLES image with real-time artificial intelligence (AI)-based detection of cancerous areas. **d** Representative CLES images alongside corresponding hematoxylin and eosin (H&E) images of non-neoplastic gastric tissue regions. The upper portion represents mucosa, the middle indicates submucosa, and the lower section denotes muscularis propria. Scale bar, 50 μm. **e** Representative CLES images and H&E images corresponding to regions of gastric cancer tissue, with the upper part illustrating adenocarcinoma and the lower part poorly cohesive carcinoma. Yellow arrow, tumor cells. Scale bar, 100 μm. **f** Schematic representation of the two-stage AI model development for interpreting CLES images. CNN convolutional neural network, ADC adenocarcinoma. **g** A representative CLES image containing gastric cancer, along with the Score-CAM for the corresponding image. CAM class activation map. Scale bar, 100 μm. **h** Representative CLES images categorized as high-confidence tumor (upper two), borderline confidence tumor (middle two), and normal (lower two) as interpreted by the AI model. Yellow arrow: tumor cells. Scale bar, 100 μm. **i** Confusion matrix and ROC curve of the AI model for detecting tumor versus normal images in the internal validation dataset. **j** Confusion matrix and ROC curve of the AI model for detecting ADC versus non-ADC in the internal validation dataset. ADC adenocarcinoma.

We employed a two-stage process in the development of CLES image-interpreting AI model (Fig. 1f). The first stage involves a convolutional neural network (CNN) (CNN 1) determining whether the input image represents a tumor. Upon identification as a tumor image, the second stage employs another CNN model (CNN 2) to classify the histologic subtype of the tumor, distinguishing between adenocarcinoma (ADC) and non-ADC (specifically, poorly cohesive carcinoma). The AI model accurately delineates the tumor area, as illustrated in the Score-class activation map (CAM)^20^ for the tumor image (Fig. 1g and Supplementary Fig. 3). Representative high-confidence images interpreted by the AI model depict the infiltration of bright cancer cell clusters, while non-neoplastic gastric tissue images, assigned a confidence score of 0, lack tumor cells (Fig. 1h). Notably, the model can distinguish dye aggregates, which reveal far-brighter variable-sized signals, from cancer cells. The model exhibits low confidence when the number of tumor cells is small, and the image is captured in a dark setting. The performance evaluation of the trained AI model in the internal validation dataset, comprising 3686 CLES images, demonstrated remarkable results with an Area under the Receiver Operating Characteristic curve (AUROC) of 1.000 for both detecting tumor images and differentiating histologic subtypes of tumors (Fig. 1i, j). As indicated in Supplementary Table 1, CNN 1 achieved an accuracy, specificity, and sensitivity of 0.964, 0.964, and 0.966, respectively in the internal validation dataset. Additionally, for histologic subtype classification (CNN 2), accuracy, specificity, and sensitivity of 0.990, 0.985, and 0.993, respectively, were achieved for ADC versus non-ADC. Despite attempts to train a multi-class classification model for a single-stage process combining tumor subtype and normal tissue, the performance was inferior compared to the two-stage process models (Supplementary Fig. 4 and Supplementary Table 2). The false-positive cases misinterpreted by the AI model encompass images featuring prominent dye aggregates, out-of-focus images, and dark images. Conversely, the false-negative cases overlooked by the AI model predominantly consist of out-of-focus images and images containing a scanty volume of tumor cells (Supplementary Figs. 5 and 6).

The standalone performance of the two-stage AI model in tumor detection was further assessed using an external validation dataset comprising 100 CLES images. The model demonstrated noteworthy proficiency in distinguishing between tumor and normal images, yielding an accuracy, specificity, and sensitivity of 0.990, 0.982, and 1.000, respectively, achieving superior performance compared to any performance by pathologists or endoscopists in previous studies (Fig. 2a and Supplementary Table 3)^6–9^. The inference time for a single image is 0.043 seconds, based on a single GPU (NVIDIA RTX A6000). Four board-certified pathologists, who underwent an educational session on CLES and its image features, also interpreted the same dataset images. However, the performances of the pathologists were significantly less effective compared to the AI model (Fig. 2b and Supplementary Table 4). To assess the value of the AI model as a complementary tool for pathologists interpreting CLES images, each pathologist initially interpreted another 100 CLES images and assessed the presence of tumor. Subsequently, the AI interpretation results were provided to them, and the revised interpretations with AI assistance were analyzed. A notable enhancement in the performance of interpreting CLES images was observed for all pathologists, with accuracy improving from 0.74 to 0.97, 0.63 to 0.85, 0.78 to 0.79, and 0.65 to 0.76 for the four pathologists, respectively (Fig. 2c and Supplementary Table 5). As depicted in the Sankey diagram, AI-assisted revisions rectified a substantial number of misinterpreted cases, although approximately 10% of the cases remained incorrect (Fig. 2d and Supplementary Fig. 7). The AI assistance also increased concordance among the pathologists, with the initial 25% agreement among all four pathologists improving to 58%. When considering concordance among more than 3 out of 4 pathologists, the initial 82% agreement was enhanced to 89% with the AI assistance (Supplementary Fig. 8).

**Fig. 2:**
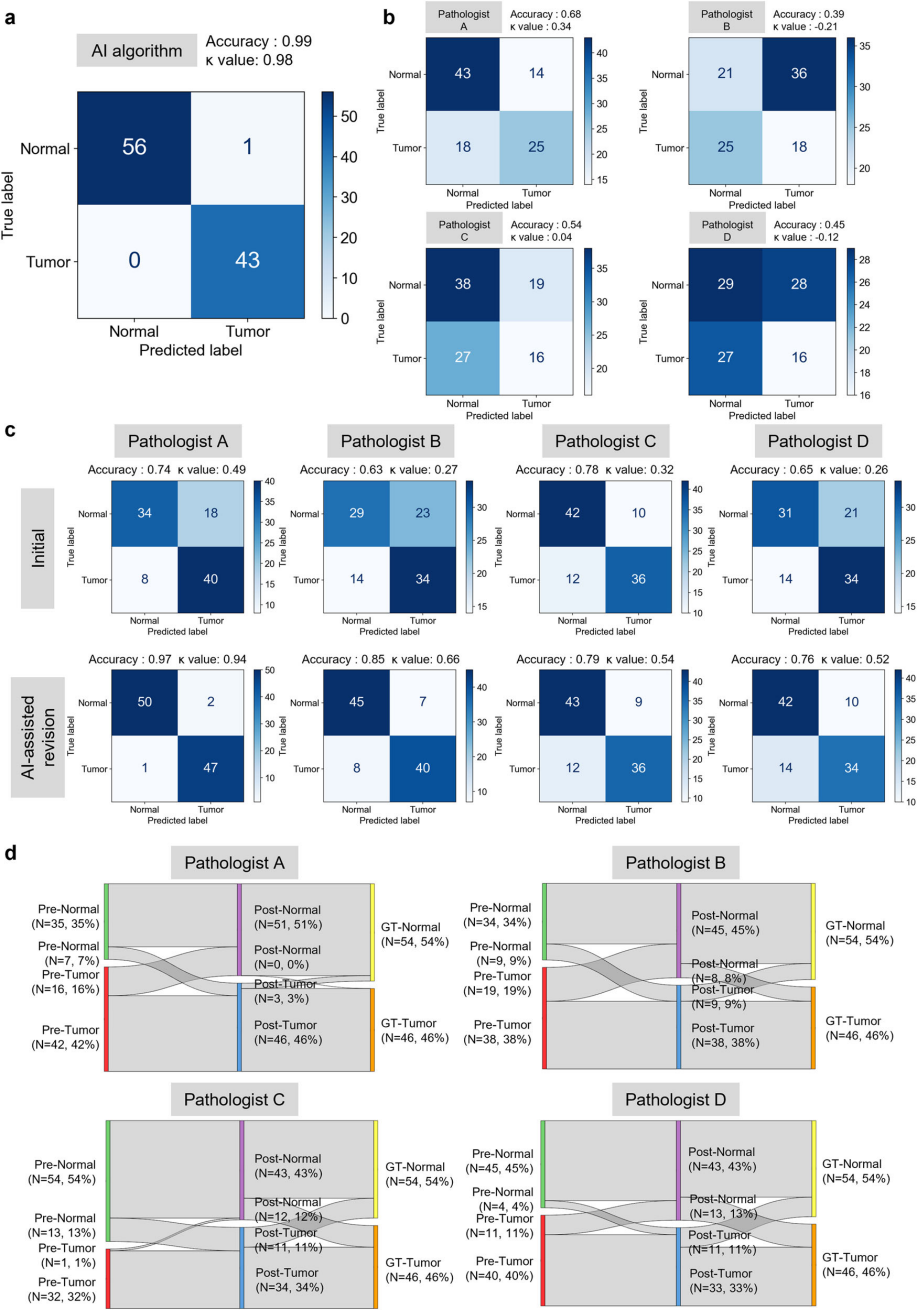
Performance validation of the artificial intelligence model and pathologists in the interpretation of confocal laser endomicroscopic images. **a** Confusion matrix illustrating the performance of the artificial intelligence (AI) model in interpreting images from the confocal laser endomicroscopic system (CLES) in the external validation dataset. **b** Confusion matrices depicting the performance of pathologists in interpreting CLES images in the external validation dataset. **c** Confusion matrices representing the performance of pathologists before (upper row) and after (lower row) AI assistance in interpreting CLES images in the external validation dataset. **d** Sankey diagrams illustrating the interpretation of CLES images by the four pathologists before and after AI assistance.

In summary, we developed a novel AI-based real-time evaluation system for gastric cancer tissue using confocal laser endomicroscopy. Employing a two-stage model eliminating the increased complexity of the CNN^21^, we first distinguished between tumor and non-neoplastic tissue and then determined the histologic subtype of gastric cancer, achieving significant performance.

Unlike conventional digital pathology AI models that operate on gigapixel pathology images, our specialized AI model processes kilopixel grayscale images, dynamically adjusting to the necessary image size. This eliminates the need to break down general pathology images into patch images, enabling tasks that previously took minutes to now be completed in less than a second. The AI model’s validation results highlight its value as a standalone tool for automated gastric cancer detection from CLES imaging. Furthermore, the significantly enhanced concordance observed among pathologists with AI assistance in interpreting CLES images underscores one of the major advantages of a real-time AI system.

This system has potential applications in intraoperative margin assessment^22^, and margin diagnosis of endoscopic submucosal dissection specimens^18^. As the real-world environment introduces various factors and artifacts into CLES images, there is the potential for decreased performance compared to our current results. Thus, fine-tuning the model using data collected from real-world settings is imperative to enhance its generalization abilities. Due to the inclusion of some CLES images from the same patients in both the training and test datasets, there exists a potential risk of overfitting of the AI model. Consequently, conducting further validation utilizing real-world datasets will be crucial to assess and mitigate this risk effectively. Also, further investigations in clinical and basic studies, exploring the relationship between CLES image features and tissue microenvironment components^23^, are warranted.

## Methods

### Dataset

A total of forty-three fresh tissue samples were obtained from patients diagnosed with gastric cancer. Tumor and normal gastric tissue samples were concurrently collected from each patient. The enrolled samples encompassed various clinicopathologic features of gastric cancer, including histological subtypes and tumor stage (Supplementary Table 6). The tissue specimens were precisely cut to dimensions of 1.0$\times$1.0$\times$0.5 cm and subsequently subjected to imaging using the CLES. Approval for this study was obtained from the Institutional Review Board (IRB) at Ajou University Medical Center, under the protocol AJIRB-BMR-KSP-22-070. Informed consent requirements were waived by the IRB due to the utilization of anonymized clinical data. The study strictly adhered to the ethical principles delineated in the Declaration of Helsinki.

### Confocal laser endomicroscopic system image acquisition

The CLES device used in this study follows a mechanical configuration described in our previous work^3^. The microscopic head and 4 mm diameter probe are positioned near the tissue, with a 488 nm light emitted from the light source (cCeLL-A 488, VPIX Medical) transmitted through an optical fiber to the tissue. The tissue, pre-applied with fluorescent dye, absorbs and emits longer-wavelength light (500–560 nm), transmitted back to the main unit through optical fibers in the probe. A stage holding the probe ensures stability during image capture. Tissue scanning utilizes a Lissajou laser-scanning pattern, allowing image acquisition up to 100μm from the tissue surface.

For tissue staining, fluorescein sodium (FNa; Sigma–Aldrich) dissolved in 30% ethanol (0.5 mg/ml) was carefully applied to the tissue sample, incubated for one minute, and rinsed with phosphate-buffered saline. After delicate cleaning to remove dye aggregates, CLES imaging captured dynamic grayscale images (1024$\times$1,024 pixels) with a field of view measuring 500$\times$500 μm. Gastric cancer and non-neoplastic tissue were scanned from the mucosa to submucosa and muscularis propria, averagely producing 500 images per tissue piece (Supplementary Fig. 1).

### Histologic evaluation of the specimen

Following the CLES imaging, tissue samples were subjected to H&E staining after fixation in 10% formalin and the creation of formalin-fixed, paraffin-embedded (FFPE) blocks. Sections of 4 μm thickness from these FFPE blocks were stained with H&E. The stained slides were then scanned at 40$\times$ magnification using the Aperio AT2 digital whole-slide scanner (Leica Biosystems). For the precise evaluation of CLES images alongside H&E-stained images, the acquired CLES images were vertically stitched from mucosa to subserosa and subsequently directly compared to the H&E images of the tissue at the same magnification (Supplementary Fig. 2). Histologic structures such as vessels or mucin pools served as landmarks for identifying the exact location. The determination of whether the CLES images from gastric tumor samples indeed contained tumor cells was facilitated through this direct comparison with the mapped H&E images. The mapping of CLES images and H&E images were conducted by experienced pathologists with gastrointestinal pathology subspecialty (S.K., and D.L.).

### Development of the artificial intelligence model

#### Preprocessing

Supplementary Table 7 outlines the acquisition of the entire 7480 tumor images and 12,928 normal images for the development and validation of the AI model. Each original image, sized at 1024$\times$1,024 pixels, was resized to 480$\times$480 pixels to align with the specifications recommended by EfficientnetV2 for CNN models^24^. These resized images underwent normalization, scaling their pixel values between 0 and 1 by dividing them by 255.

#### Classification model development

EfficientnetV2, a model achieving state-of-the-art performance in Imagenet 2021, and renowned for its high processing speed, was utilized for developing the tumor classification model (CNN 1) and the tumor subtype classification model (CNN 2)^24^. To determine the model capacity in terms of the number of layers and filters among the hyperparameters, we compared the performance of two variants of the EfficientNetV2 model: EfficientNetV2-S (with approximately 22 million parameters) and EfficientNetV2-M (with approximately 54 million parameters) after training. The EfficientNetV2-S model was selected due to its superior performance. Experimentation revealed that when employing high learning rates such as 0.1 or 0.001, overfitting occurred early in the epochs, leading to a bias towards either tumor or normal classes. Hence, a lower learning rate of 0.0001 was employed to encourage the model to converge gradually during training.

We conducted 5-fold cross-validation of the two models, allocating 80% of the entire dataset for training and the remaining 20% for testing. To achieve a balanced ratio between tumor and normal classes during training and mitigate overfitting caused by class imbalance, we down-sampled the normal image set to align with the number of tumor images. This down-sampling process involved random sampling with a fixed seed value. As a result, among the preprocessed images, 5984 tumor images and 5984 normal images (in a 1:1 ratio) were utilized in training the AI model. The final performance was calculated as the average and standard deviation of the accuracy, sensitivity, and specificity among the folds. Each model is trained for 50 epochs in each fold with batch size 16, AdamW optimizer with a default parameter, and cross-entropy loss function. In order to derive better generalization performance in the training process, data augmentation techniques such as flip and rotation were applied. As depicted in Fig. 1f, we developed a two-stage process that distinguishes the tumor and the subtype of the CLES image with the two CNN models mentioned above. (1) In the first stage, the input CLES image is determined as tumor or normal by CNN 1. In the sigmoid output of the CNN 1 model for the image, it is indicated as a tumor if it is greater than 0.5, or if it is less, it is indicated as normal. (2) In the second stage, CNN 2 classifies the tumor subtype of the tumor-determined CLES image. As in the first stage, if it is greater than 0.5 in the CNN 2 sigmoid output of the input tumor image, it is classified as ADC, or if it is less, then classified as non-ADC. To determine the threshold value as 0.5, we compared the performance of the model across different thresholds by considering precision and F1 score, as shown in Supplementary Tables 8 and 9. The optimal threshold for each fold was determined using Youden’s index^25^, resulting in values of 0.506, 0.508, 0.523, 0.573, and 0.546, respectively. 1496 tumor images and 2586 normal images were utilized for the test in each fold. Despite the slight enhancement of performance with the thresholds calculated from Youden’s index, we decided to utilize the median value of the sigmoid function, 0.5, as the default threshold because the true positive rate and true negative rate exhibit variability depending on the chosen threshold, potentially introducing bias towards specific classes. Following the model development, 3686 images were utilized for the internal validation of the model performance.

#### Activation map analysis

The activation map of the CNN 1 model was created using Score-CAM to determine whether the CNN 1 model trained the imaging features related to the tumor normally. Score-CAM removes dependence on the slope by acquiring the weight of each activation map through the forward pass score for the target class, and the final result is obtained by a linear combination of the weight and the activation map, so it shows an improved result compared to the previous class activation map^20^. As shown in Fig. 1g, in the activation map, the area activated in the CNN 1 prediction is shown in red.

### External validation of the standalone performance of the artificial intelligence model and pathologists’ performance

The standalone performance assessment of the two-stage AI model in detecting tumor images involved the utilization of 43 tumor images and 57 normal images from 14 patient samples. Metrics such as sensitivity, specificity, and accuracy for detecting tumor images were calculated. Concurrently, four experienced pathologists independently analyzed the same validation dataset comprising 100 CLES images, determining whether each image contained tumor cells. Prior to the task, they underwent group training for interpreting CLES images conducted by an experienced gastrointestinal pathologist (S.K.) well-acquainted with CLES. In addition to the training, the four pathologists were provided with 200 CLES images and their corresponding H&E images for further study. Sensitivity, specificity, accuracy, and Cohen’s kappa value, in comparison to the ground truth data, were assessed.

A separate dataset of 100 CLES images, including 46 tumor images and 54 normal images from 15 patient samples, was presented to the pathologists in a distinct session. After their initial interpretation regarding the presence of tumor cells in each image, the AI interpretation results were disclosed to the pathologists for assistance, allowing them to revise their analytical results. Cohen’s kappa value was utilized to show inter-observer agreement. Sensitivity, specificity, and accuracy were also calculated both before and after the AI assistance to comprehensively evaluate the impact of AI support on the pathologists’ performance.

### Statistical analysis

AUROC was used to evaluate the performance of the AI models. Cohen’s kappa was applied to evaluate the concordance of tumor/normal distinction between the ground truth and the interpreted result. All statistical analyses were carried out using Python 3.8 and R version 4.0.3 software (R Foundation for Statistical Computing).

### Reporting summary

Further information on research design is available in the Nature Research Reporting Summary linked to this article.

### Supplementary information

Supplementary material

Supplementary video 1

Reporting summary

## Data Availability

The data in this study are available from the corresponding author upon reasonable request.

## Code availability

The program was developed using Python programming language (version 3.8). The models are implemented using PyTorch v1.10 (available at https://github.com/pytorch/pytorch) and Scikit-learn v1.0.2 (available at https://github.com/scikit-learn/scikit-learn/blob/main/sklearn/model_selection/_split.py). The activation map analysis process is implemented using Score-CAM (open source implementations available online, https://github.com/frgfm/torch-cam). The data augmentation transformations are implemented using Albumentations v1.3.1 (https://github.com/albumentations-team/albumentations), OpenCV Python v4.7.0.68 (https://github.com/opencv/opencv-python). Mathematical operations are implemented using Numpy v1.23.4 (https://github.com/numpy/numpy). For any questions regarding the replication of results, the corresponding author can be contacted.
