# Supplementary material for: Artificial intelligence-based real-time histopathology of gastric cancer using confocal laser endomicroscopy
Source: NPJ Precis Oncol. 2024 Jun 14;8:131. doi: 10.1038/s41698-024-00621-x (PMC11178780; doi:10.1038/s41698-024-00621-x)
Supplement: Supplementary file 1 — Supplementary material [file 41698_2024_621_MOESM1_ESM.pdf]

Supplementary Information for
Artificial Intelligence-based Real-Time Histopathology of Gastric Cancer Using Confocal Laser Endomicroscopy

Haeyon Cho[1*], Damin Moon[2*], So Mi Heo[3*], Jinah Chu[4*], Hyunsik Bae[5,6], Sangjoon Choi[1], Yubin Lee[2], Dongmin Kim[2], Yeonju Jo[7], Kyuyoung Kim[7], Kyungmin Hwang[7], Dakeun Lee[3,8†], Heung-Kook Choi[2†], Seokhwi Kim[3,8†]

[1]Department of Pathology, Asan Medical Center, Ulsan University Medical School, Seoul, Republic of Korea

[2]JLK Artificial Intelligence R&D Center, Seoul, Republic of Korea

[3]Department of Pathology, Ajou University School of Medicine, Suwon, Republic of Korea

[4]Department of Pathology, Kangbuk Samsung Hospital, Sungkyunkwan University School of Medicine, Seoul, Republic of Korea

[5]Department of Pathology and Translational Genomics, Samsung Medical Center, Sungkyunkwan University School of Medicine, Seoul, Republic of Korea

[6]Pathology center, Seegene Medical Foundation, Seoul, Republic of Korea

[7]VPIX Medical Inc., Daejeon, Republic of Korea

[8]Department of Biomedical Sciences, Ajou University Graduate School of Medicine, Suwon, Republic of Korea

[*]These authors contributed equally to this work.

[†] Co-corresponding authors: Seokhwi Kim, Heung-Kook Choi, Dakeun Lee.

Supplementary Fig. 1 Operation user interface of artificial intelligence-based confocal laser endomicroscopic system. a Adjusting focus process. **b** Image capture process. **c** Image location map (yellow box). **d** Captured image without activation map overlay. **e** Captured image with activation map overlay. Red box, button for switching to activation map. Reprinted with permission from VPIX Medical, Inc.

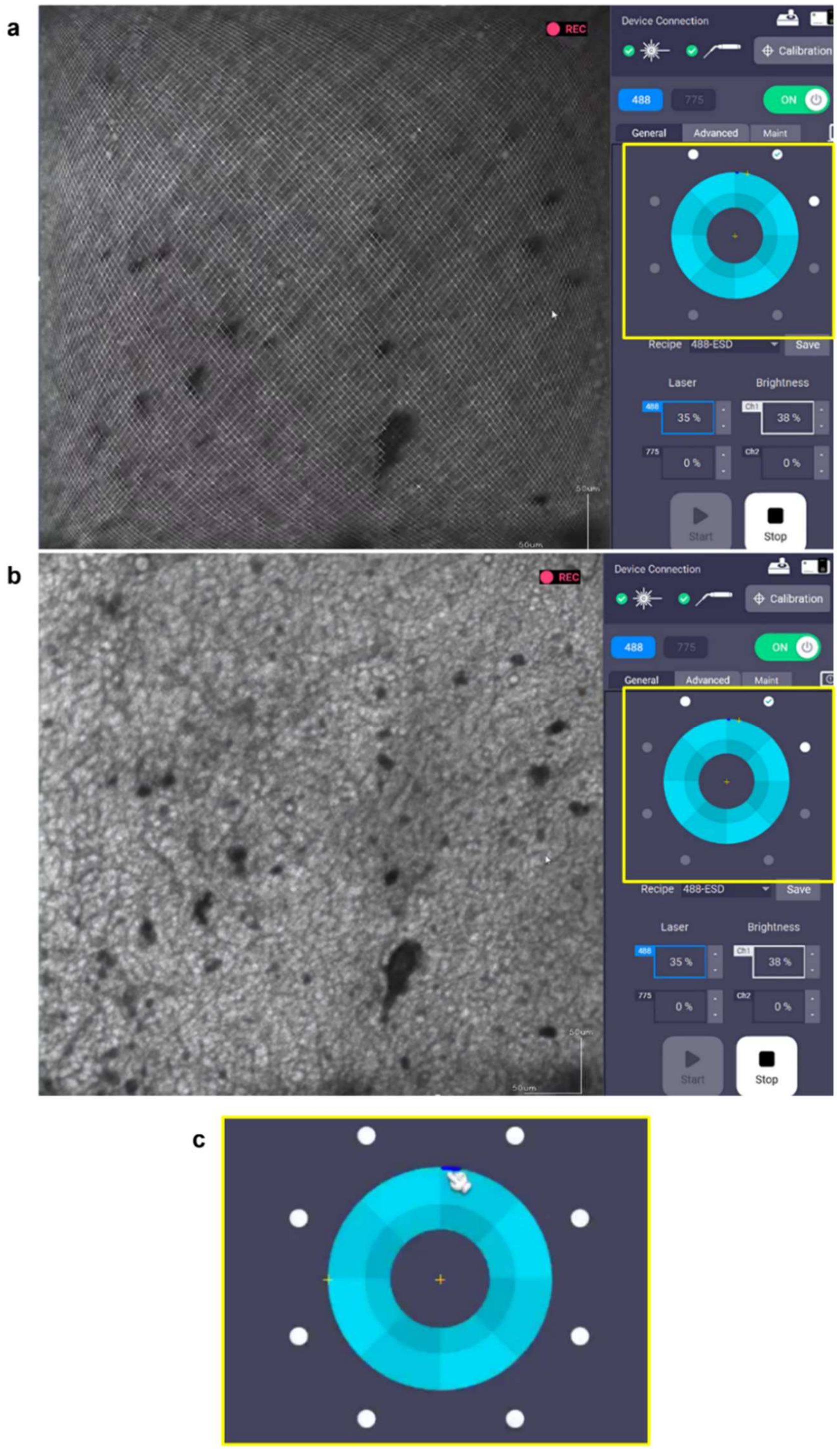

d

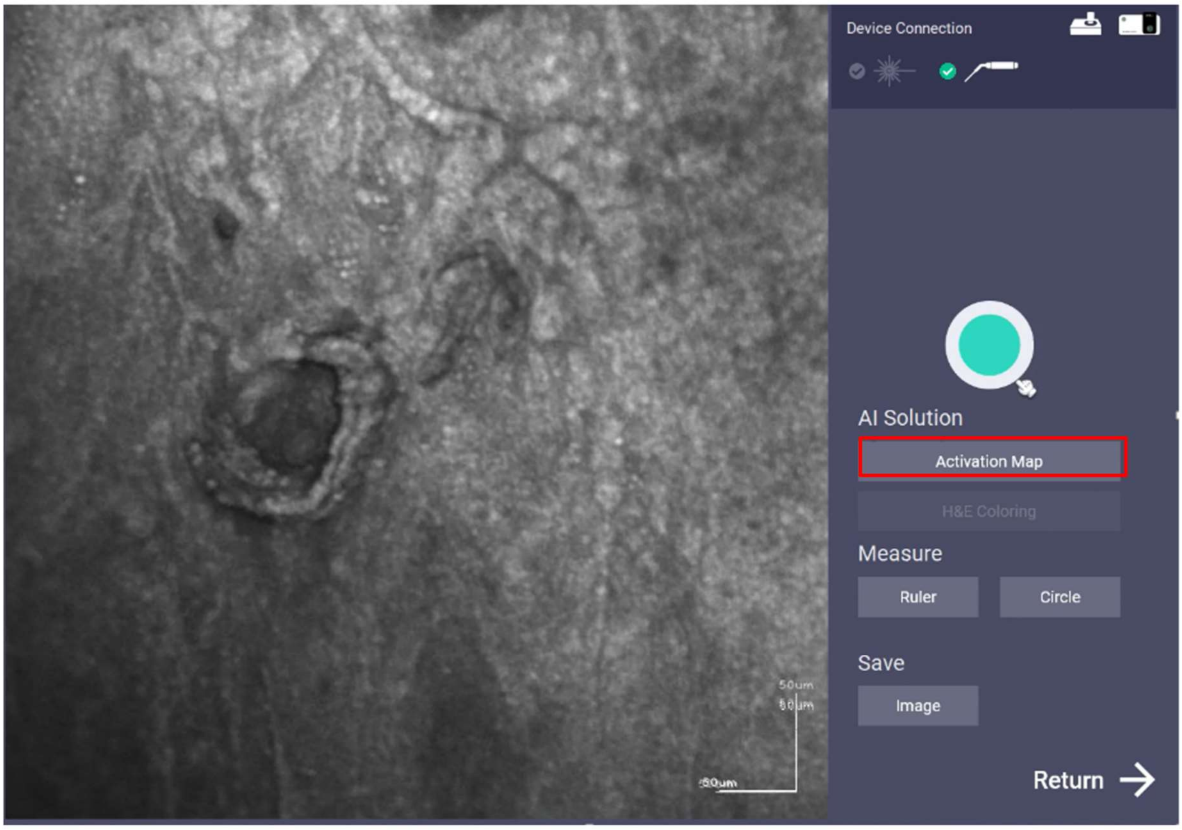
Device Connection
AI Solution
Activation Map
H&E Coloring
Measure
Ruler
Circle
Save
Image
Return
50um
50um

e

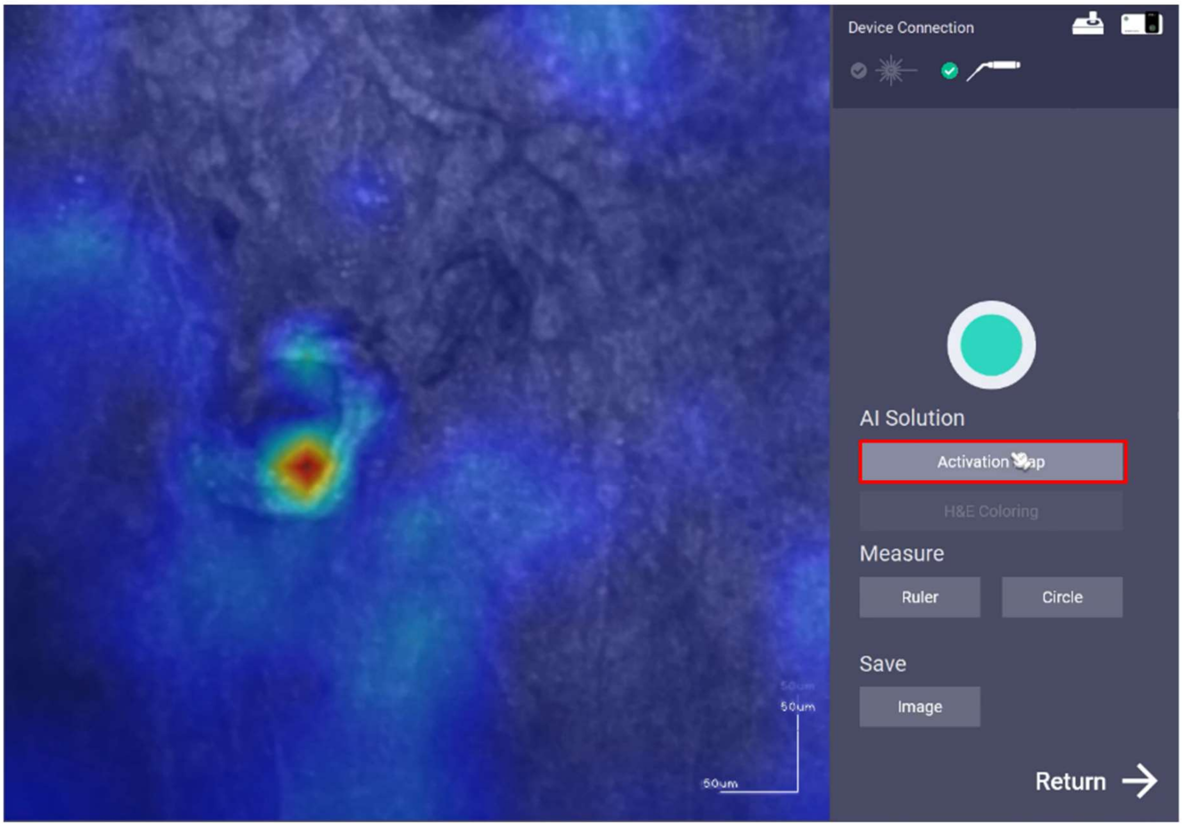
Device Connection
AI Solution
Activation Map
H&E Coloring
Measure
Ruler
Circle
Save
Image
Return
50um
50um

Supplementary Fig. 2 Representative confocal laser endomicroscopic system images and hematoxylin and eosin-stained histologic images of samples mapping for the comparison. **a** Representative stitched confocal laser endomicroscopic system (CLES) image (left) and hematoxylin and eosin (H&E)-stained histologic images (right) of normal gastric tissue. Yellow arrow, a vessel in submucosa which can be used as a landmark. **b** Representative stitched CLES image (left) and H&E images (right) of gastric cancer tissue. Red arrowhead, mucin pool which can be used as a landmark. Yellow arrowhead, tumor glands. Scale bar, 500μm.

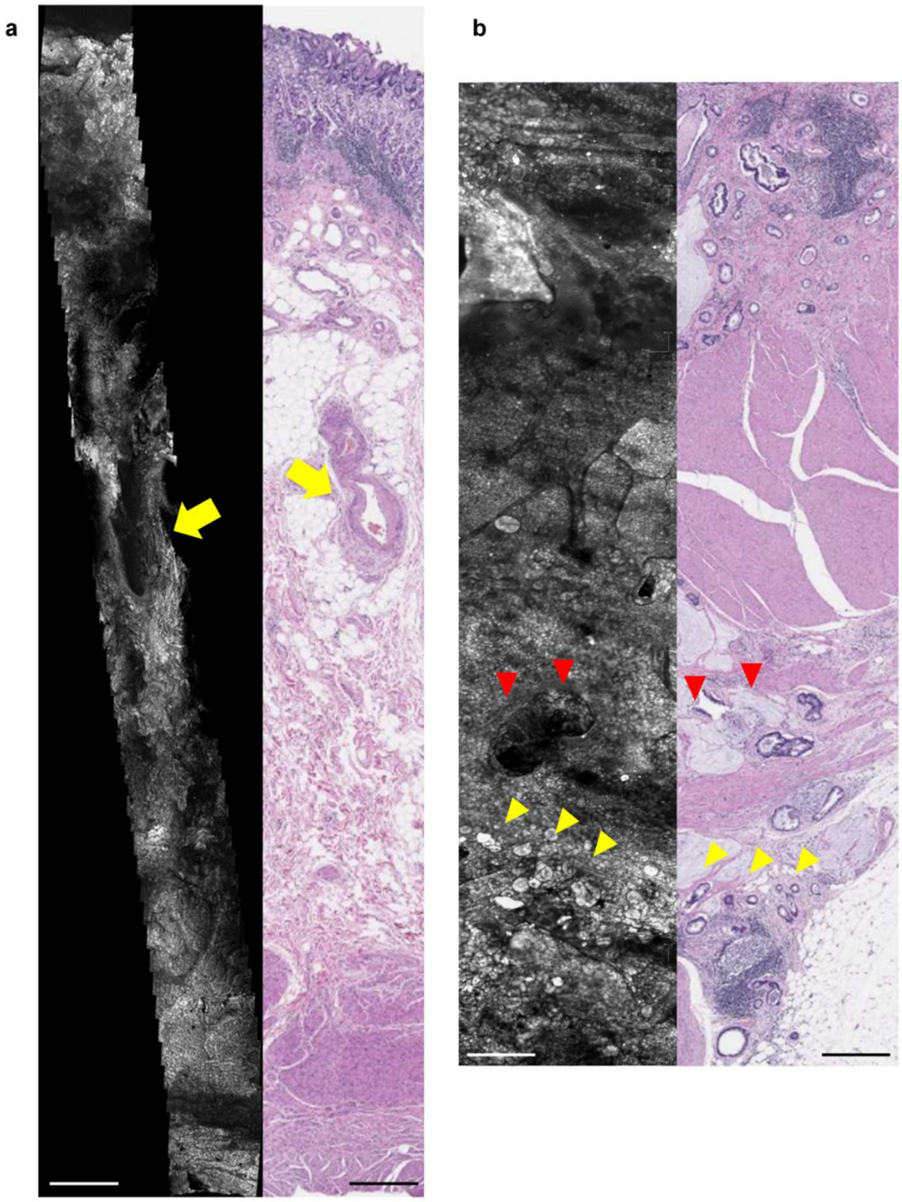

Supplementary Fig. 3 Representative confocal laser endomicroscopic system (CLES) images and Score-CAM for gastric adenocarcinoma (ADC) and gastric non-adenocarcinoma. Scale bar, 50μm.

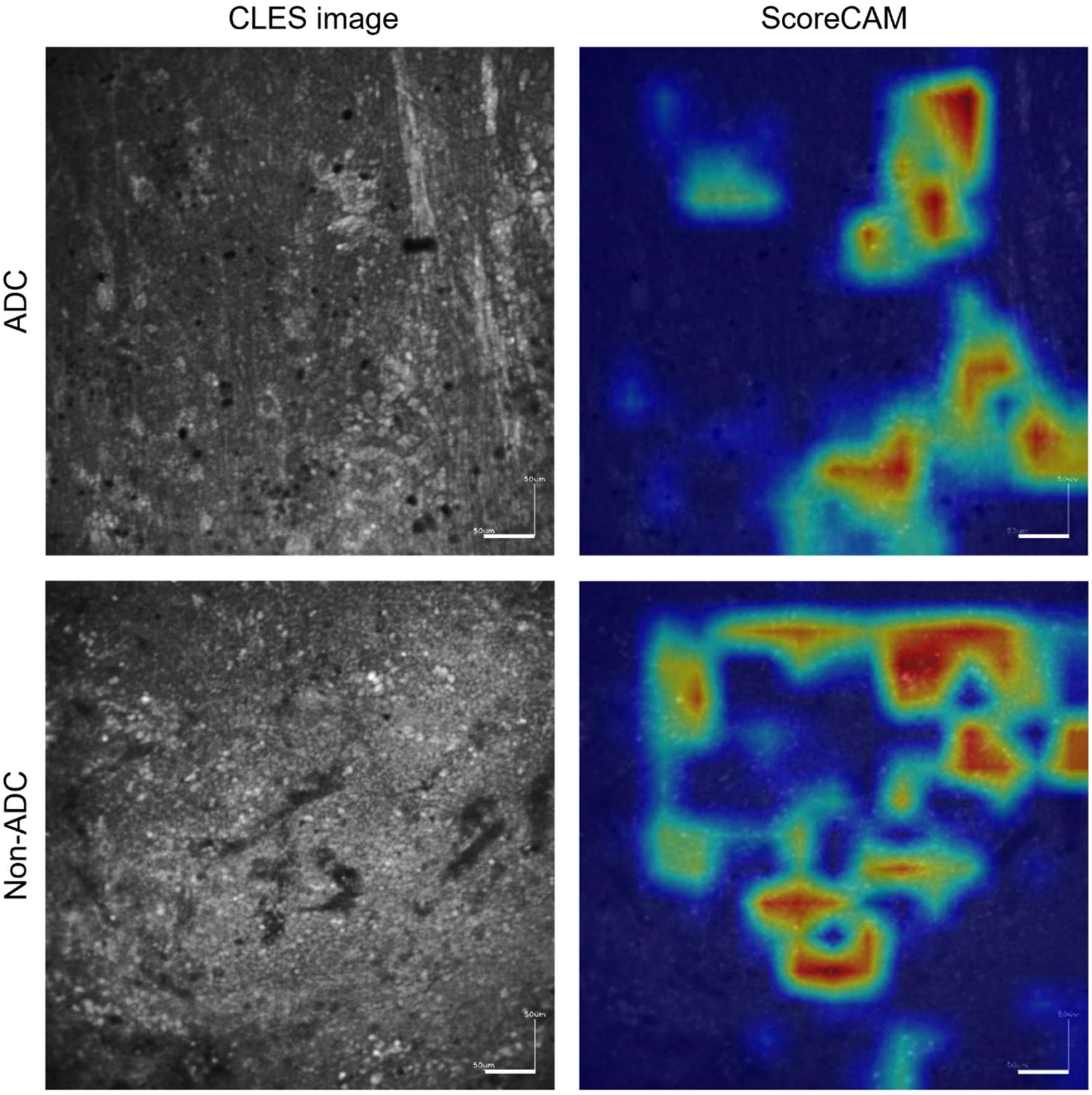

Supplementary Fig. 4 Development of the single-stage artificial intelligence model of confocal laser endomicroscopic system images to differentiate normal gastric tissue, gastric adenocarcinoma, and gastric non-adenocarcinoma. a Schematic representation of the single-stage artificial intelligence (AI) model's development for interpreting confocal laser endomicroscopic system (CLES) images. CNN, convolutional neural network, ADC, adenocarcinoma. **b** Confusion matrix of the AI model for detecting ADC versus non-ADC versus normal images in the internal validation dataset. **c** ROC curve of the AI model for detecting ADC versus non-ADC versus normal images in the internal validation dataset. Class 0, normal images, class 1, ADC, class 2, non-ADC.

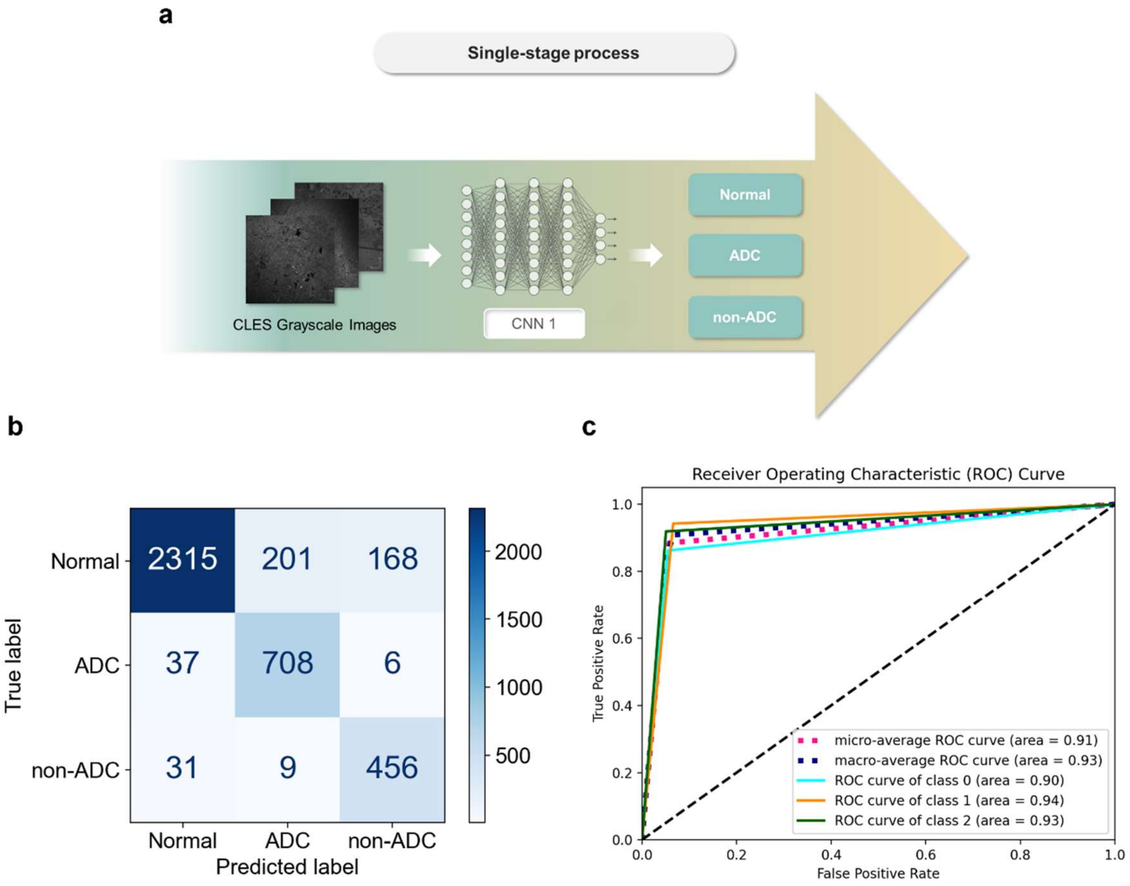

Supplementary Fig. 5 False positive and false negative confocal laser endomicroscopic system images identified by an artificial intelligence-based interpretation system in the two-stage model, along with proposed explanations. Scale bar, 50μm.

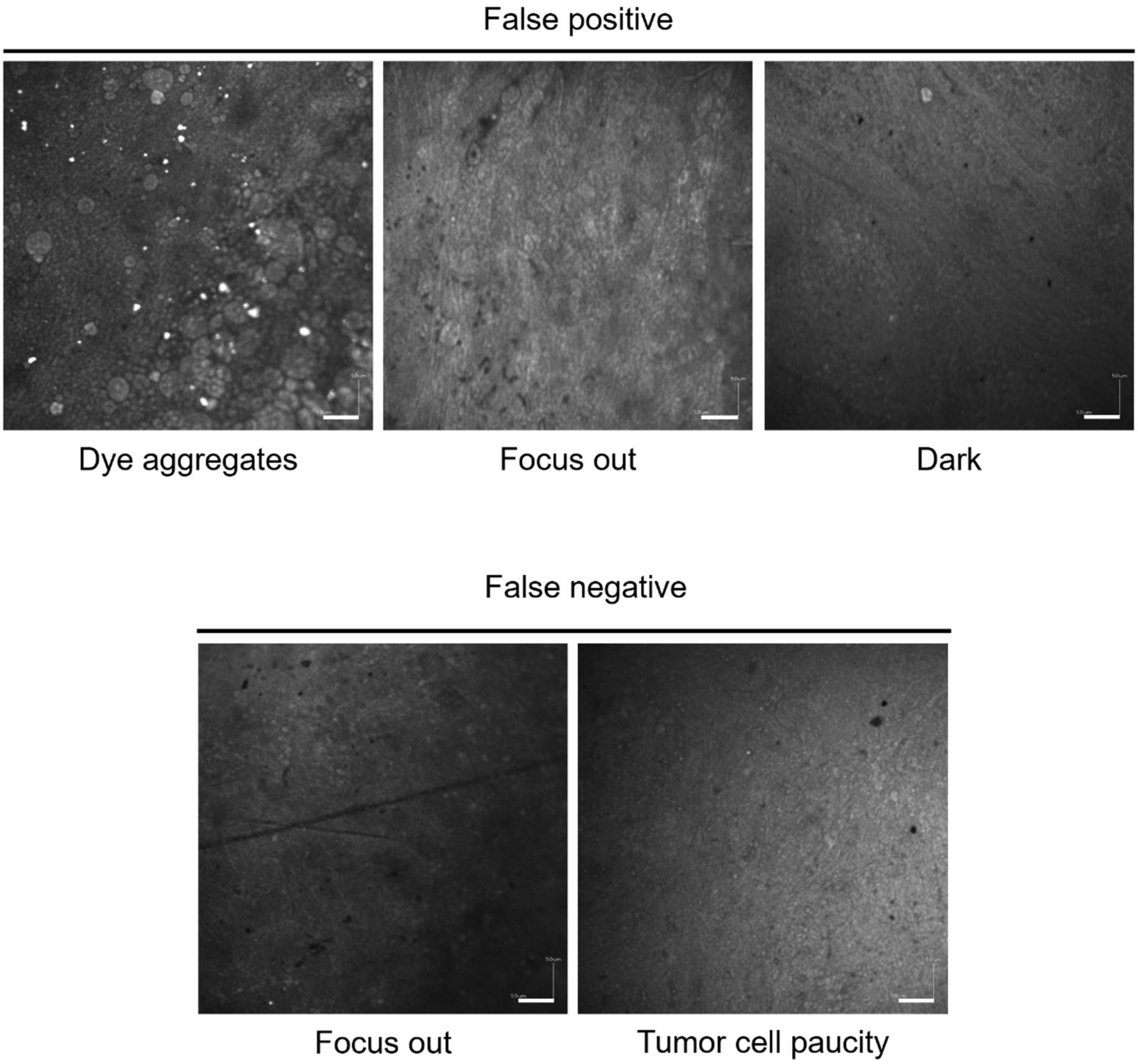

Supplementary Fig. 6 False positive confocal laser endomicroscopic system images identified by an artificial intelligence-based interpretation system in the single-stage model. a-b Normal sample image misinterpreted to adenocarcinoma (ADC). **c-d** Normal sample image misinterpreted to non-ADC. CLES, confocal laser endomicroscopic system. CAM, class activation map. Scale bar, 50μm.

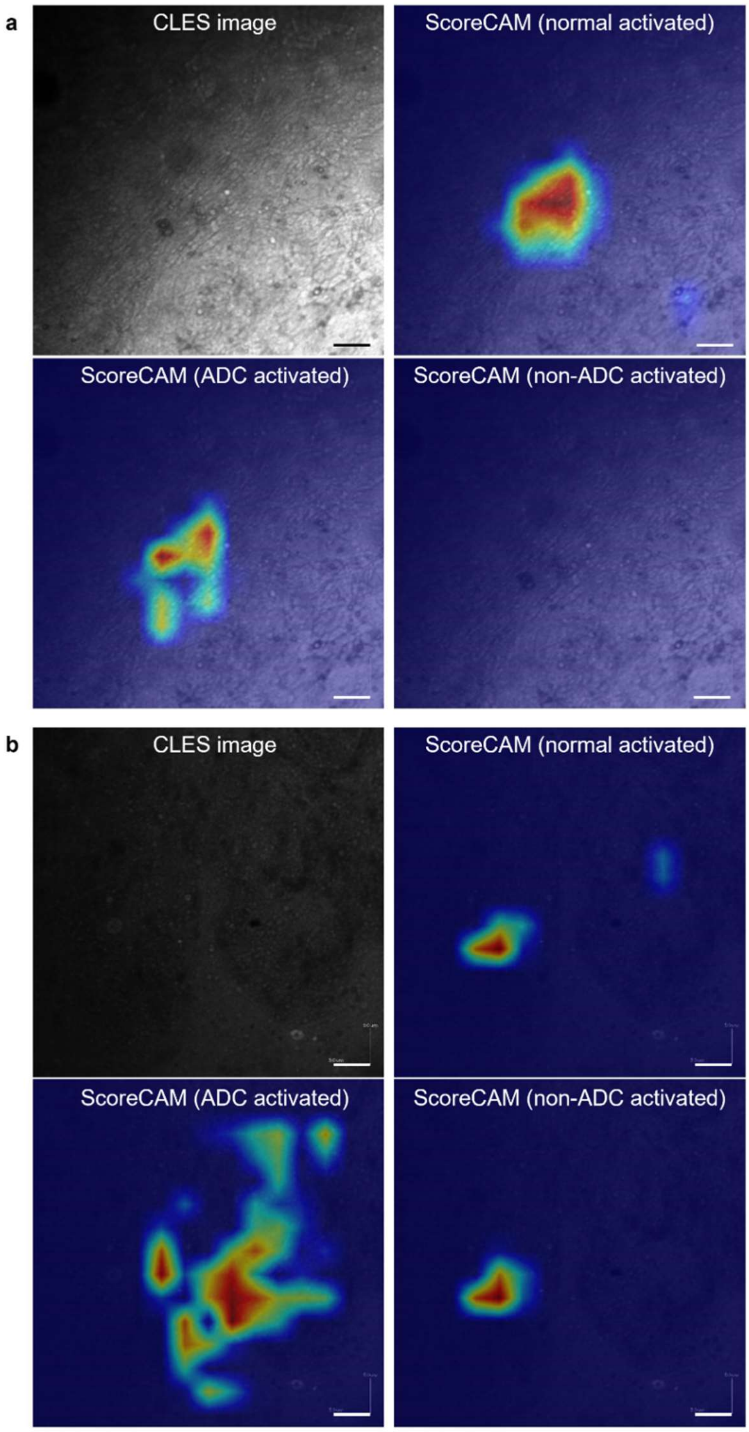

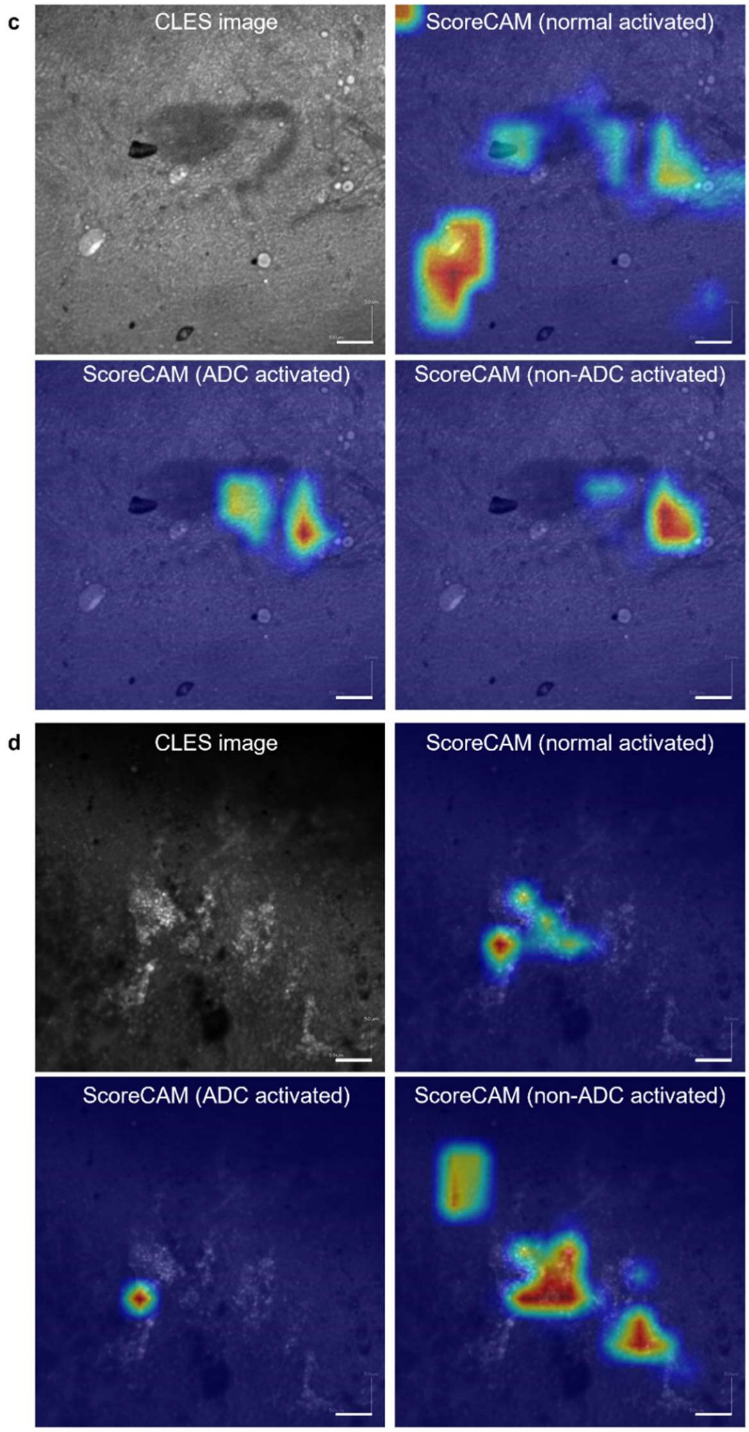
c
CLES image
ScoreCAM (normal activated)
ScoreCAM (ADC activated)
ScoreCAM (non-ADC activated)
d
CLES image
ScoreCAM (normal activated)
ScoreCAM (ADC activated)
ScoreCAM (non-ADC activated)

Supplementary Fig. 7 Sankey diagram for all four pathologists' interpretation of confocal laser endomicroscopic system images pre- and post-artificial intelligence assistance.

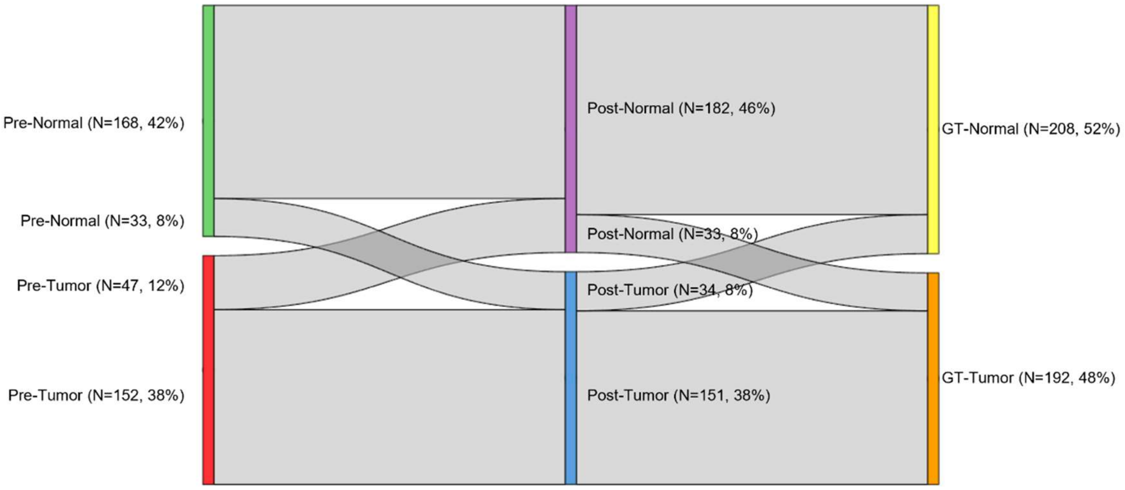

Supplementary Fig. 8 Agreement in the interpretation of confocal laser endomicroscopic image by pathologists before and after the artificial intelligence assistance. a Agreement among all four pathologist cases. **b** Concordance among cases where more than three out of four pathologists are in agreement.

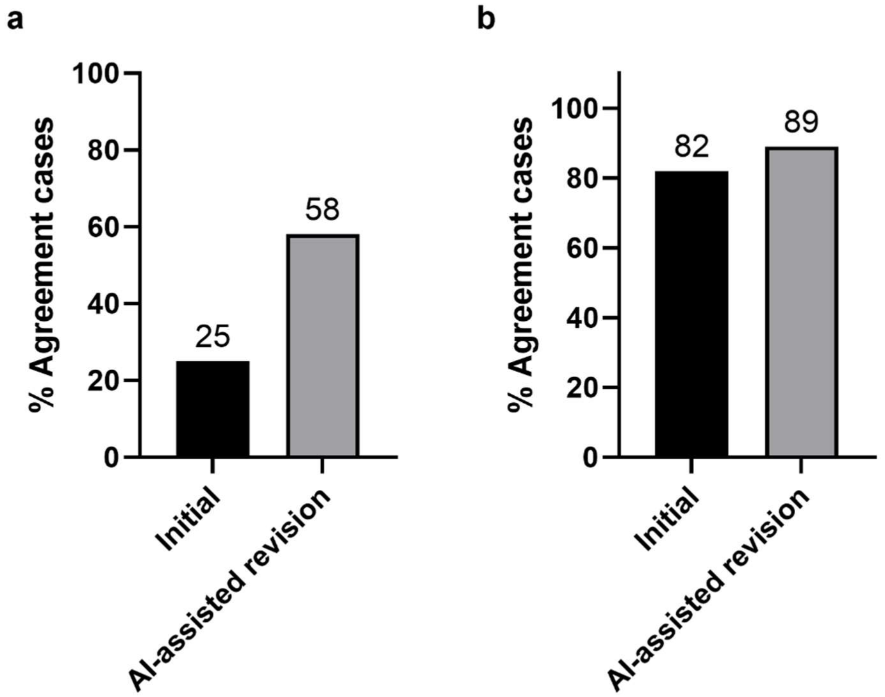

Supplementary Table 1. Classification performances of the two-stage artificial intelligence model for interpreting confocal laser endomicroscopic system images in the internal validation dataset.

	**Accuracy**	**Specificity**	**Sensitivity**
Tumor vs Normal	0.964±0.005	0.964±0.007	0.966±0.005
ADC vs non-ADC	0.990±0.004	0.985±0.009	0.993±0.002

ADC, adenocarcinoma.

Supplementary Table 2. Classification performance of the single-stage artificial intelligence model for interpreting confocal laser endomicroscopic system images in the internal validation dataset.

	Accuracy	Specificity	Sensitivity
Normal vs ADC vs non-ADC	0.832±0.020	0.840±0.025	0.913±0.014

ADC, adenocarcinoma.

Supplementary Table 3. Diagnostic performances of the artificial intelligence model in this study for the external validation dataset and previous literature.

	Sample number	Accuracy	Specificity	Sensitivity	Interpreter
Bok *et al.,* 2013	46	0.917	N/A	N/A	Experienced endoscopist
Kitabatake *et al.,* 2006	27	0.952	0.976	0.864	Two pathologists
Gong *et al.,* 2015	82	0.919	0.935	0.900	Two pathologists
Li *et al.,* 2019	33	N/A	0.818	0.778	Three experienced endoscopist
AI model in this study	43	0.990	0.982	1.000	AI model

N/A, not available, AI, artificial intelligence.

Supplementary Table 4. Performances of the AI model and pathologists for the external validation dataset.

	Accuracy	Specificity	Sensitivity	Cohen's Kappa
AI model	0.990	0.982	1.000	0.980
Pathologist A	0.680	0.754	0.581	0.340
Pathologist B	0.390	0.368	0.420	-0.206
Pathologist C	0.540	0.667	0.372	0.040
Pathologist D	0.450	0.509	0.372	-0.119

Supplementary Table 5. Performances of the pathologists with AI-assisted interpretation for the external validation dataset.

	Initial interpretation				AI-assisted revision			
	Accuracy	Specificity	Sensitivity	Cohen's Kappa	Accuracy	Specificity	Sensitivity	Cohen's Kappa
Pathologist A	0.740	0.654	0.833	0.487	0.970	0.961	0.980	0.940
Pathologist B	0.630	0.558	0.708	0.268	0.850	0.865	0.833	0.658
Pathologist C	0.780	0.808	0.750	0.322	0.790	0.827	0.750	0.536
Pathologist D	0.650	0.596	0.708	0.261	0.760	0.808	0.708	0.515

Supplementary Table 6. Clinicopathologic characteristics of the patient samples.

Characteristics	N=43
Age	
Median [Range]	65 [41, 86]
Sex	
Male	31 (72.1%)
Female	12 (27.9%)
Lauren's classification	
Intestinal	12 (27.9%)
Diffuse	15 (34.9%)
Indeterminate	9 (20.9%)
Mixed	4 (9.3%)
Not defined	3 (7.0%)
Histologic type	
Adenocarcinoma (ADC)	24 (55.8%)
Poorly cohesive carcinoma (PCC)	14 (32.6%)
Mixed ADC and PCC	4 (9.3%)
Others	1 (2.3%)
Tumor location	
Upper 1/3	7 (16.3%)
Middle 1/3	11 (25.6%)
Lower 1/3	19 (44.2%)
Upper and middle 1/3	1 (2.3%)
Middle and lower 1/3	1 (2.3%)
Entire stomach	4 (9.3%)
Pathologic T stage	
T1	14 (32.6%)
T2	4 (9.3%)
T3	4 (9.3%)
T4	21 (48.8%)
Pathologic N stage	
N0	21 (48.8%)
N1	4 (9.3%)
N2	5 (11.6%)
N3	13 (30.2%)
AJCC staging group (8th edition)	
I	16 (37.2%)
II	7 (16.3%)
III	20 (46.5%)

Supplementary Table 7. The number of confocal laser endomicroscopic system images utilized for the development of an artificial intelligence-based interpretation model.

	**Number of tumor images**	**Number of normal images**
Total data	7,480	12,928
Training data in each fold (normal data down-sampled)	5,984	5,984
Test data in each fold	1,496	2,586
	**Number of ADC images**	**Number of non-ADC images**
Total data	4,250	3,230

ADC, adenocarcinoma.

Supplementary Table 8. The performance of the AI model in applying threshold 0.5 and the threshold determined from Youden's index in each fold.

Metric	Fold 1 Threshold		Fold 2 Threshold		Fold 3 Threshold		Fold 4 Threshold		Fold 5 Threshold	
	0.5	0.506	0.5	0.508	0.5	0.523	0.5	0.573	0.5	0.546
Accuracy	0.958	0.958	0.965	0.965	0.974	0.974	0.961	0.963	0.963	0.965
Specificity	0.956	0.957	0.967	0.967	0.974	0.976	0.957	0.960	0.964	0.967
Sensitivity	0.962	0.961	0.961	0.960	0.973	0.972	0.970	0.968	0.962	0.962
Precision	0.923	0.924	0.940	0.941	0.954	0.956	0.924	0.930	0.935	0.941
F1 Score	0.955	0.955	0.962	0.962	0.972	0.972	0.958	0.960	0.960	0.962

Supplementary Table 9. The performance of the AI model in applying threshold 0.5 and the threshold determined from Youden's index (average value of each fold).

	Accuracy	Specificity	Sensitivity	Precision	F1 score
Tumor vs Normal Threshold 0.5	0.964 ±0.005	0.964 ±0.007	0.966 ±0.005	0.935 ±0.011	0.961 ±0.006
Tumor vs Normal Threshold Youden's index	0.965 ±0.005	0.965 ±0.007	0.965 ±0.005	0.938 ±0.011	0.962 ±0.006

Supplementary Video 1. Operation example of artificial intelligence-based confocal laser endomicroscopic system.

Included separately as a video file.
