# Supplementary material for: Artificial intelligence-based real-time histopathology of gastric cancer using confocal laser endomicroscopy
Source: NPJ Precis Oncol. 2024 Jun 14;8:131. doi: 10.1038/s41698-024-00621-x (PMC11178780; doi:10.1038/s41698-024-00621-x)
Supplement: Supplementary file 3 — Reporting summary [file 41698_2024_621_MOESM3_ESM.pdf]

nature portfolio

Corresponding author(s): Seokhwi Kim

Last updated by author(s): Apr 11, 2024

Reporting Summary

Nature Portfolio wishes to improve the reproducibility of the work that we publish. This form provides structure for consistency and transparency in reporting. For further information on Nature Portfolio policies, see our Editorial Policies and the Editorial Policy Checklist.

Statistics

For all statistical analyses, confirm that the following items are present in the figure legend, table legend, main text, or Methods section.

n/a	Confirmed	
☐	☒	The exact sample size (n) for each experimental group/condition, given as a discrete number and unit of measurement
☐	☒	A statement on whether measurements were taken from distinct samples or whether the same sample was measured repeatedly
☐	☒	The statistical test(s) used AND whether they are one- or two-sided<br>*Only common tests should be described solely by name; describe more complex techniques in the Methods section.*
☒	☐	A description of all covariates tested
☐	☒	A description of any assumptions or corrections, such as tests of normality and adjustment for multiple comparisons
☐	☒	A full description of the statistical parameters including central tendency (e.g. means) or other basic estimates (e.g. regression coefficient) AND variation (e.g. standard deviation) or associated estimates of uncertainty (e.g. confidence intervals)
☐	☒	For null hypothesis testing, the test statistic (e.g. F, t, r) with confidence intervals, effect sizes, degrees of freedom and P value noted<br>*Give P values as exact values whenever suitable.*
☒	☐	For Bayesian analysis, information on the choice of priors and Markov chain Monte Carlo settings
☒	☐	For hierarchical and complex designs, identification of the appropriate level for tests and full reporting of outcomes
☐	☒	Estimates of effect sizes (e.g. Cohen's d, Pearson's r), indicating how they were calculated

Our web collection on statistics for biologists contains articles on many of the points above.

Software and code

Policy information about availability of computer code

Data collection	The program was developed using Python programming language (version 3.8). The models are implemented using PyTorch v1.10 (available at https://github.com/pytorch/pytorch) and Scikit-learn v1.0.2 (available at https://github.com/scikit-learn/scikit-learn/blob/main/sklearn/model_selection/_split.py). Clinical dataset was collected using R software v4.0.3.
Data analysis	The program was developed using Python programming language (version 3.8). The models are implemented using PyTorch v1.10 (available at https://github.com/pytorch/pytorch) and Scikit-learn v1.0.2 (available at https://github.com/scikit-learn/scikit-learn/blob/main/sklearn/model_selection/_split.py). The activation map analysis process is implemented using ScoreCAM (open source implementations available online, https://github.com/frgfm/torch-cam). The data augmentation transformations are implemented using Albumentations v1.3.1 (https://github.com/albumentations-team/albumentations), OpenCV Python v4.7.0.68 (https://github.com/opencv/opencv-python). Mathematical operations are implemented using Numpy v1.23.4 (https://github.com/numpy/numpy). Data was analyzed using Python 3.7 and R version 4.0.3 software.

For manuscripts utilizing custom algorithms or software that are central to the research but not yet described in published literature, software must be made available to editors and reviewers. We strongly encourage code deposition in a community repository (e.g. GitHub). See the Nature Portfolio guidelines for submitting code & software for further information.

Data

Policy information about availability of data

All manuscripts must include a data availability statement. This statement should provide the following information, where applicable:

- Accession codes, unique identifiers, or web links for publicly available datasets
- A description of any restrictions on data availability
- For clinical datasets or third party data, please ensure that the statement adheres to our policy

We have provided a full data availability statement in the manuscript that the data in this study are available from the corresponding author upon reasonable request.

Research involving human participants, their data, or biological material

Policy information about studies with human participants or human data. See also policy information about sex, gender (identity/presentation), and sexual orientation and race, ethnicity and racism.

Reporting on sex and gender	Biological samples were obtained from any participants of both sex, and sex or gender were not a factor in the sampling collection. Among the 43 human samples in this study, 31 were from male and 12 were from female.
Reporting on race, ethnicity, or other socially relevant groupings	The race of participants are Asians for all samples.
Population characteristics	Supplementary Table 6 describes the population characteristics in this study.
Recruitment	Recruitment was conducted according to the institutional review board policy of the institution.
Ethics oversight	The study protocol was approved by the institutional review board of the Ajou University Medical Center (AJIRB-BMR-KSP-22-070).

Note that full information on the approval of the study protocol must also be provided in the manuscript.

Field-specific reporting

Please select the one below that is the best fit for your research. If you are not sure, read the appropriate sections before making your selection.

☒ Life sciences ☐ Behavioural & social sciences ☐ Ecological, evolutionary & environmental sciences

For a reference copy of the document with all sections, see nature.com/documents/nr-reporting-summary-flat.pdf

Life sciences study design

All studies must disclose on these points even when the disclosure is negative.

Sample size	43 gastric sample from patients
Data exclusions	This study excluded cases that did not meet the data collection criteria.
Replication	Not applicable
Randomization	Not applicable
Blinding	Images of both tumor tissues and normal tissues were provided to pathologists without indicating whether they originated from tumor or normal tissue samples.

Reporting for specific materials, systems and methods

We require information from authors about some types of materials, experimental systems and methods used in many studies. Here, indicate whether each material, system or method listed is relevant to your study. If you are not sure if a list item applies to your research, read the appropriate section before selecting a response.

Materials & experimental systems

n/a	Involved in the study
☒	☐ Antibodies
☒	☐ Eukaryotic cell lines
☒	☐ Palaeontology and archaeology
☒	☐ Animals and other organisms
☒	☐ Clinical data
☒	☐ Dual use research of concern
☒	☐ Plants

Methods

n/a	Involved in the study
☒	☐ ChIP-seq
☒	☐ Flow cytometry
☒	☐ MRI-based neuroimaging

Plants

Seed stocks	Not applicable
Novel plant genotypes	Not applicable
Authentication	Not applicable
